# Survival Outcomes and Machine Learning-Based Prediction of 12-Month Mortality in Glioblastoma Before and During the COVID-19 Pandemic: A SEER Population-Based Study

**DOI:** 10.3390/medicina62061169

**Published:** 2026-06-16

**Authors:** Yasemin Adalı, Ömer Emin Çınar, Ümit Akın Dere

**Affiliations:** 1 Centre for Public Health, School of Medicine, Dentistry and Biomedical Sciences, Queen’s University Belfast, Belfast BT7 1NN, UK; 2 Department of Epidemiology, Centre for Public Health, Pamukkale University Faculty of Medicine, Denizli 20070, Türkiye; 3 School of Electronics, Electrical Engineering and Computer Science, Queen’s University Belfast, Belfast BT7 1NN, UK; 4 Department of Computer Engineering, Faculty of Engineering and Architecture, Recep Tayyip Erdogan University, Rize 53100, Türkiye; 5 Department of Neurosurgery, Pamukkale University Faculty of Medicine, Denizli 20070, Türkiye

**Keywords:** glioblastoma, COVID-19, cancer epidemiology, SEER, survival analysis, machine learning

## Abstract

*Background and Objectives*: The COVID-19 pandemic disrupted cancer diagnosis and treatment pathways worldwide. Glioblastoma is an aggressive primary brain malignancy requiring timely multimodal care. This study evaluated survival outcomes among glioblastoma patients diagnosed before and during the COVID-19 pandemic and prepared a dataset for machine learning-based prediction of 12-month mortality. *Materials and Methods*: Patients aged ≥20 years diagnosed with glioblastoma between 2018 and 2021 were identified from the SEER database using ICD-O-3 histology codes 9440/3, 9441/3, and 9442/3. Patients were categorized as pre-COVID period (2018–2019) or COVID period (2020–2021). OS and CSS were evaluated using Kaplan–Meier curves, log-rank tests, and Cox regression models. Machine learning models predicted 12-month all-cause mortality using registry variables. *Results*: The final cohort included 9914 patients; 4819 were diagnosed pre-COVID and 5095 during COVID. Median OS was 11 months pre-COVID and 10 months during COVID; 12-month OS was 44.3% and 41.2%, respectively. Median CSS was 11 months in both periods; 12-month CSS was 46.9% and 44.1%, respectively. COVID-period diagnosis was modestly associated with poorer OS (adjusted HR 1.050, 95% CI 1.006–1.095, *p* = 0.025) and CSS (adjusted HR 1.048, 95% CI 1.003–1.095, *p* = 0.035). Machine learning models showed moderate discrimination for 12-month mortality prediction. *Conclusions*: Glioblastoma patients diagnosed during the COVID period had modestly poorer OS and CSS in conventional survival analyses; however, competing-risk analysis did not show a significant association with cancer-specific death. Registry-based machine learning models provided moderate 12-month mortality prediction, supporting their potential utility for population-level prognostic assessment.

## 1. Introduction

Glioblastoma is the most aggressive primary malignant tumor of the central nervous system in adults and continues to be associated with poor survival despite advances in neurosurgical, radiotherapeutic, and systemic treatment strategies [1]. The 2021 World Health Organization classification of central nervous system tumors has increasingly emphasized integrated histomolecular diagnosis, particularly the distinction of glioblastoma as an adult type diffuse glioma with IDH-wildtype molecular features [2]. However, large-scale population-based cancer registry studies frequently rely on histology and topography-based definitions, which remain valuable for evaluating temporal trends, treatment patterns, and survival outcomes across broad health care systems [3].

Standard treatment for newly diagnosed glioblastoma generally involves maximal safe surgical resection when feasible, followed by radiotherapy with concomitant and adjuvant temozolomide [4]. Although this multimodal approach has improved outcomes compared with radiotherapy alone, the prognosis remains limited, and glioblastoma remains a highly time-sensitive malignancy [5]. Therefore, disruptions in diagnostic pathways, surgical access, radiotherapy delivery, chemotherapy administration, or multidisciplinary neuro oncology care may have meaningful consequences, particularly during periods of health system strain.

The COVID-19 pandemic caused substantial disruption to cancer services worldwide. Systematic reviews have documented delays and interruptions across cancer screening, diagnosis, surgery, radiotherapy, systemic therapy, follow-up, and supportive care. Riera et al. identified multiple categories of pandemic-related delays and disruptions affecting cancer diagnosis, treatment, and general health services, while more recent evidence has continued to show that these disruptions were global and clinically relevant [6,7]. In the United States and other high-income settings, population-based studies have also reported reductions in cancer diagnoses and measurable declines in short-term survival among patients diagnosed during the pandemic period [8,9].

Evidence regarding the impact of the COVID-19 pandemic on glioma and glioblastoma care remains limited and somewhat heterogeneous. A systematic review and meta-analysis by Azab et al. evaluated glioma management during the pandemic and highlighted the need to understand how pandemic conditions affected neurosurgical and oncological care pathways [10]. Subsequent institutional and regional studies reported variable findings. For example, Mischkulnig et al. found that the pandemic did not negatively affect overall survival among surgically treated high-grade glioma patients at their institution, whereas Chahal et al. evaluated treatment patterns and outcomes among grade 4 glioma patients treated with radiation during the pandemic in British Columbia [11,12]. These findings suggest that the effect of the pandemic may have differed according to health-care setting, patient selection, treatment prioritization, and regional service organization.

More recent studies have specifically examined glioblastoma and malignant brain tumor care during the pandemic. Neff et al. evaluated the impact of the COVID-19 pandemic on treatment patterns in glioblastoma and reported that national-level treatment patterns could be assessed using large-scale registry data [13]. Karamani et al. examined tumor size, treatment patterns, and survival in neuro-oncology during the COVID-19 pandemic and reported no significant survival difference between pre-COVID and COVID periods in the overall cohort and glioblastoma subgroup [14]. Similarly, Qin et al. reported that glioblastoma survival remained stable across pre- and post-COVID periods, while advanced age, bilateral tumors, and passive treatment remained important prognostic factors [15]. Together, these studies indicate that glioblastoma services may have been relatively protected in some settings because of the urgent nature of the disease; however, population-level survival effects remain incompletely defined.

In contrast, population-based studies in other malignancies have increasingly demonstrated that the pandemic period was associated with changes in diagnosis, treatment, and short-term survival. Hong et al. reported that 1-year relative survival among patients diagnosed with cancer in the United States declined during the early pandemic period, with particularly marked reductions for selected cancer types [9]. Tang et al. used SEER data to examine six major cancer sites and found that patients diagnosed in 2020 were more likely to present with high-grade tumors, with evidence of adverse changes in treatment and prognosis for several cancers [16]. More recently, Burus et al. reported worse 1-year cause-specific survival among patients diagnosed with cancer in 2020 and 2021 compared with those diagnosed before the pandemic, suggesting measurable short-term survival consequences of pandemic-related cancer care disruption [8]. These registry-based findings support the need for cancer-specific analyses in aggressive tumors such as glioblastoma.

Alongside conventional survival analysis, machine learning approaches have increasingly been applied to glioblastoma prognostication. Machine learning and deep learning models may integrate demographic, clinical, tumor-related, treatment, imaging, and molecular variables to estimate survival or short-term mortality risk [17]. A recent systematic review by Poursaeed et al. summarized the growing use of machine learning and deep learning methods for glioblastoma survival prediction and emphasized that model performance depends strongly on the type and quality of input variables [18]. SEER-based machine learning studies have also shown that routinely available registry variables can be used to predict glioblastoma survival outcomes, although such models require careful interpretation because registry datasets lack several clinically important molecular and performance-status variables [19].

Therefore, this population-based SEER study aimed to evaluate survival outcomes among adult glioblastoma patients diagnosed before and during the COVID-19 pandemic. Specifically, we compared overall survival and cancer-specific survival between patients diagnosed in 2018–2019 and those diagnosed in 2020–2021, adjusting for demographic, tumor-related, socioeconomic, and treatment-related factors. In addition, we developed machine learning models to predict 12-month all-cause mortality and compared model performance across pre-COVID, COVID, and combined cohorts. By integrating conventional survival analysis with machine learning-based short-term mortality prediction, this study sought to clarify whether diagnosis during the COVID-19 period was associated with measurable survival disadvantage in glioblastoma and whether routinely available registry variables could provide clinically relevant prognostic information. The machine learning component was included to complement the survival analysis by evaluating whether the same routinely available registry variables could provide short-term mortality prediction across pre-COVID and COVID-period cohorts, thereby exploring whether pandemic-era diagnosis was accompanied by changes in registry-based prognostic patterns.

## 2. Materials and Methods

### 2.1. Data Source and Study Population

This population-based retrospective cohort study used data from the Surveillance, Epidemiology, and End Results (SEER) Program of the National Cancer Institute. Data were extracted using SEER*Stat software version 9.0.42.2 from the “Incidence-SEER Research Data, 17 Registries, November 2024 Submission, released April 2025” database. Patients diagnosed with glioblastoma between 2018 and 2021 were identified. This study period was selected to compare survival outcomes before and during the COVID-19 pandemic.

Glioblastoma cases were identified using the International Classification of Diseases for Oncology, Third Edition (ICD-O-3) histology codes 9440/3, 9441/3, and 9442/3, corresponding to glioblastoma, NOS; giant cell glioblastoma; and gliosarcoma, respectively. Primary tumor sites were restricted to the brain using ICD-O-3 topography codes C71.0-C71.9.

Patients were eligible if they had malignant disease, positive microscopic or histological diagnostic confirmation, and were aged 20 years or older at diagnosis. Patients younger than 20 years were excluded because the SEER age recode variable groups patients aged 15–19 years together, preventing the precise separation of patients aged 18–19 years from minors. Cases identified only through a death certificate or autopsy were excluded. To reduce potential bias related to prior malignancies, only first primary malignant tumors were retained using the SEER sequence number variable.

The diagnosis period was categorized as the pre-COVID period (2018–2019) and the COVID period (2020–2021). The initial SEER extraction identified 13,151 glioblastoma records diagnosed between 2018 and 2021. After excluding patients aged <20 years (n = 125), death certificate only or autopsy only cases (n = 99), non-first primary malignancies (n = 2386), and cases without microscopic or histological confirmation (n = 627), the final analytic cohort included 9914 adult patients with glioblastoma. A separate 12-month machine learning cohort included 9798 eligible patients after excluding those without sufficient follow-up for 12-month mortality classification.

### 2.2. Variables

The primary exposure variable was the diagnosis period, categorized as the pre-COVID period (2018–2019) and the COVID period (2020–2021). Demographic and socioeconomic variables included age at diagnosis, sex, race, marital status, rural-urban continuum code, and county-level median household income adjusted to 2024 inflation. Tumor-related variables included primary tumor site, histologic subtype, laterality, grade variables, and diagnostic confirmation. Treatment-related variables included surgery of the primary site, radiotherapy, chemotherapy, surgery/radiotherapy sequence, reason for no cancer-directed surgery, and time from diagnosis to treatment.

### 2.3. Outcomes

The primary survival outcomes were overall survival (OS) and cancer-specific survival (CSS). OS was defined as the time from diagnosis to death from any cause. Patients who were alive at the study cutoff were censored. CSS was defined as the time from diagnosis to death attributable to glioblastoma, based on the SEER cause-specific death classification. Patients who were alive or died from causes other than glioblastoma were censored for CSS analysis.

For the machine learning component, the primary prediction outcome was 12-month all-cause mortality, defined as death from any cause within 12 months after diagnosis. Patients who survived beyond 12 months were classified as non-events. Patients who were alive but had less than 12 months of potential follow-up were excluded from the 12-month mortality prediction dataset.

### 2.4. Statistical Analysis

Baseline demographic, tumor-related, socioeconomic, and treatment characteristics were summarized according to diagnosis period. Categorical variables were reported as frequencies and percentages. Comparisons between patients diagnosed in the pre-COVID and COVID periods were performed using Pearson’s chi-square test. Survival outcomes were assessed separately for overall survival (OS) and cancer-specific survival (CSS). Kaplan–Meier methods were used to estimate survival functions according to diagnosis period, and differences between survival curves were compared using the log-rank test. Cox proportional hazards regression models were used to estimate hazard ratios (HRs) and 95% confidence intervals (CIs) for the association between diagnosis period and survival outcomes. Univariable Cox regression was first performed to assess the crude association between diagnosis period and OS or CSS. Multivariable Cox regression models were subsequently constructed to adjust for potential confounders, including age group, sex, race, marital status, primary tumor site, histologic subtype, laterality, surgery, radiotherapy, chemotherapy, rural-urban status, and median household income. The proportional hazards assumption was assessed using Schoenfeld residual-based tests. All epidemiological analyses were conducted using Stata version 16.1 (StataCorp LLC, College Station, TX, USA). A two-sided *p*-value < 0.05 was considered statistically significant.

### 2.5. Machine Learning Analysis

A supervised binary classification framework was developed to predict 12-month all-cause mortality among adult glioblastoma patients across three temporally defined datasets: the pre-COVID-19 period, the COVID-19 pandemic period, and a combined longitudinal cohort. The binary outcome was defined as death within 12 months of diagnosis (1 = dead, 0 = alive), derived exclusively from the survival months and vital status fields. Both variables were strictly excluded from the predictor set to prevent data leakage.

The feature space included demographic characteristics (age, sex, race/ethnicity), diagnosis period, tumor site, histology, laterality, rural-urban classification, and median household income. Clinical intervention variables such as surgery, chemotherapy, and radiation (administration and timing) were also incorporated.

All categorical variables were encoded using one-hot encoding prior to model training. Missing or unrecorded values in categorical predictors were assigned to an explicit “Unknown” category rather than being imputed or excluded, thereby preserving the full analytic sample while transparently representing data limitations. Continuous variables were standardized (zero mean, unit variance) before training Logistic Regression and Support Vector Machine models, which are sensitive to feature scale; tree-based models (Random Forest, Gradient Boosting) were trained on unscaled features.

Within each cohort, data were partitioned into training (80%) and held-out test (20%) sets using stratified random splitting to preserve the class distribution of the outcome in both subsets. Final performance metrics were computed exclusively on the held-out test set, which was not used at any point during model training or hyperparameter selection.

To evaluate whether predictive models generalize across time, a prerequisite for real-world clinical deployment, models trained on the pre-COVID-19 cohort were additionally applied to the COVID-19 cohort as a temporally independent external validation set. This design intentionally mirrors a prospective deployment scenario and provides a more conservative and clinically meaningful estimate of generalizability than within-period random splitting alone.

Because 12-month mortality constituted the minority class in this registry-based cohort, class imbalance was addressed during training rather than through resampling. Class weights were set inversely proportional to class frequency for Logistic Regression and Gradient Boosting; the class_weight = “balanced” parameterization was applied to Random Forest and SVM. This approach was preferred over synthetic oversampling (e.g., SMOTE) to avoid generating artificial instances in a clinical registry where the multivariate feature covariance structure may not support meaningful interpolation.

Four supervised learning algorithms were implemented: Logistic Regression (L2 regularization), Random Forest, Gradient Boosting, and Support Vector Machine (radial basis function kernel). These were selected to represent a spectrum from linear to complex non-linear decision boundaries and to allow comparison of interpretability versus predictive complexity.

Hyperparameters were optimized within each training set using 5-fold stratified cross-validation, with AUC-ROC as the selection criterion. The search grids were as follows: regularization strength *C* (0.01, 0.1, 1, 10) for Logistic Regression and SVM; number of estimators (100, 300, 500), maximum depth (3, 5, 10), and minimum samples per leaf (1, 5, 10) for Random Forest; and learning rate (0.01, 0.05, 0.1), number of estimators (100, 300, 500), and maximum depth (3, 5, 7) for Gradient Boosting. Final models were retrained on the complete training set using the selected hyperparameter configuration before evaluation on the held-out test set.

All cross-validation was conducted using stratified k-fold splitting (k = 5) restricted to the training partition. Stratification ensured proportional class representation across folds, yielding unbiased tuning estimates despite the imbalanced outcome. Cross-validation was used solely for hyperparameter selection and was never applied to the held-out test set or the temporal validation cohort.

Model performance on the held-out test set was assessed using a comprehensive suite of metrics computed separately for each class and as weighted averages. For each class k (Alive or Dead), Sensitivity (Recall) is the proportion of true k instances correctly predicted as k; Specificity is the proportion of true non-k instances correctly predicted as non-k (i.e., sensitivity of the complementary class); Precision is the proportion of predicted k instances that are truly k; and the F1-Score is the harmonic mean of Precision and Recall for class k. Overall Accuracy is the proportion of all instances correctly classified. The weighted-average F1-Score weights each class by its support (number of true instances), accounting for class imbalance. AUC-ROC summarizes discrimination across all classification thresholds. The Matthews Correlation Coefficient (MCC) was used as the primary summary metric, as it is robust to class imbalance and reflects both sensitivity and specificity simultaneously across both classes via a single balanced coefficient.

SHAP (SHapley Additive exPlanations) analysis was conducted on the best-performing model within each cohort to quantify individual feature contributions to predicted mortality risk. This enabled a granular, period-stratified comparison of the clinical drivers of short-term mortality before and during the COVID-19 pandemic.

All analyses were implemented in Python 3.12 (scikit-learn) within the Google Colab environment. Source code is publicly available via a GitHub repository detailed in the Supplementary Materials.

## 3. Results

### 3.1. Patient Selection and Baseline Characteristics

**Figure 1:**
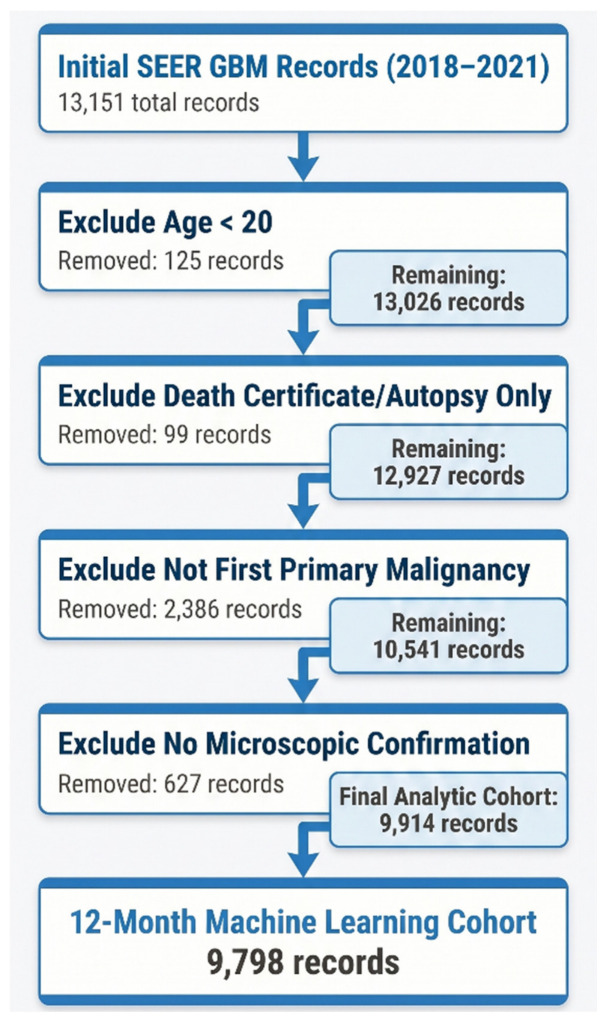
Patient selection flowchart for the SEER-based glioblastoma cohort.

A total of 13,151 glioblastoma records diagnosed between 2018 and 2021 were initially identified from the SEER database. After excluding patients aged <20 years, cases identified only through death certificate or autopsy, non-first primary malignancies, and cases without microscopic or histological confirmation, the final analytic cohort included 9914 adult patients with glioblastoma. The 12-month machine learning cohort included 9798 eligible patients after excluding those without sufficient follow-up for 12-month mortality classification (Figure 1).

Among the 9914 patients included in the final analytic cohort, 4819 (48.6%) were diagnosed during the pre-COVID period and 5095 (51.4%) during the COVID period. Overall, most patients were male (58.8%) and White (86.4%). The most common age group was 60–69 years (33.0%), followed by 70–79 years (25.6%) and 50–59 years (22.5%). The most frequent tumor sites were the frontal lobe (30.3%), temporal lobe (26.4%), and parietal lobe (15.6%). Most tumors were classified as glioblastoma, NOS (97.1%), while GBM variants, including giant cell glioblastoma and gliosarcoma, accounted for 2.9% of cases.

**Figure 2:**
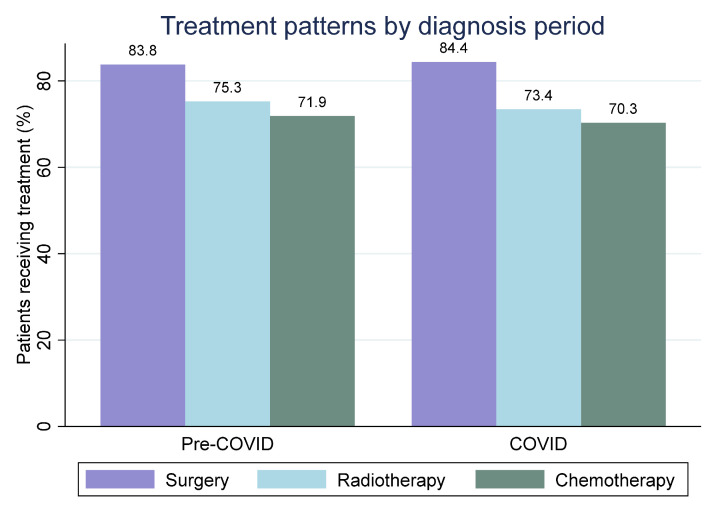
Treatment patterns among glioblastoma patients diagnosed before and during the COVID-19 pandemic.

Baseline demographic and tumor characteristics were broadly similar between the pre-COVID and COVID groups. There were no statistically significant differences by sex, race, marital status, tumor site, or histology. However, the age group distribution differed modestly between diagnosis periods (*p* = 0.034). Treatment patterns also showed some differences: the distribution of surgical procedures differed significantly between periods (*p* = 0.018), and radiotherapy use was slightly lower in the COVID period compared with the pre-COVID period (73.4% vs. 75.3%, *p* = 0.038). Chemotherapy use was also numerically lower during the COVID period, although this difference did not reach statistical significance (70.3% vs. 71.9%, *p* = 0.087) (Table 1 and Figure 2).

### 3.2. Survival Outcomes by Diagnosis Period

**Figure 3:**
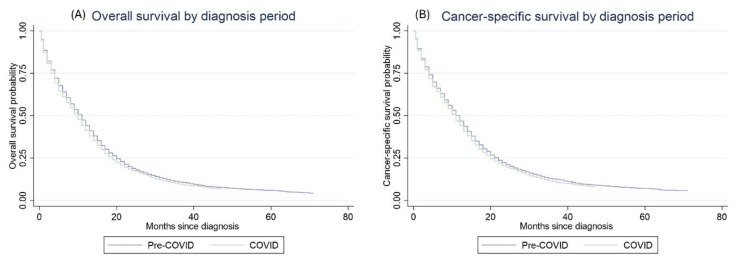
Overall (**A**) and cancer-specific (**B**) survival among glioblastoma patients diagnosed before and during the COVID-19 pandemic.

Kaplan–Meier analysis demonstrated a statistically significant difference in overall survival between diagnosis periods. Patients diagnosed during the COVID period had poorer overall survival compared with those diagnosed during the pre-COVID period (log-rank *p* = 0.0032) (Figure 3A). Cancer-specific survival was also significantly worse among patients diagnosed during the COVID period compared with the pre-COVID period (log-rank *p* = 0.0057) (Figure 3B). Median OS was 11 months in the pre-COVID period and 10 months in the COVID period. The 12-month OS probability was 44.3% in the pre-COVID period and 41.2% in the COVID period, corresponding to an absolute difference of approximately 3.1 percentage points. Median CSS was 11 months in both periods, while the 12-month CSS probability was 46.9% in the pre-COVID period and 44.1% in the COVID period, corresponding to an absolute difference of approximately 2.8 percentage points.

### 3.3. Multivariable Cox Regression for Overall Survival

In the multivariable Cox regression model for overall survival, diagnosis during the COVID period remained independently associated with worse overall survival compared with diagnosis during the pre-COVID period (adjusted HR: 1.050, 95% CI: 1.006–1.095, *p* = 0.025) (Table 2). Increasing age was strongly associated with poorer overall survival, with the highest risk observed among patients aged ≥80 years compared with those aged <50 years (adjusted HR: 3.123, 95% CI: 2.817–3.463, *p* < 0.001). Male sex was also associated with increased all-cause mortality (adjusted HR: 1.130, 95% CI: 1.083–1.180, *p* < 0.001). Treatment variables showed strong associations with overall survival. Compared with no surgery, local excision/biopsy, subtotal resection, radical/total resection, extended resection, and surgery NOS/other were all associated with improved overall survival. Radical/total resection showed one of the strongest associations with improved OS (adjusted HR: 0.581, 95% CI: 0.544–0.621, *p* < 0.001). Receipt of radiotherapy (adjusted HR: 0.560, 95% CI: 0.519–0.605, *p* < 0.001) and chemotherapy (adjusted HR: 0.525, 95% CI: 0.487–0.564, *p* < 0.001) were also independently associated with better overall survival. Gliosarcoma histology was associated with poorer OS compared with glioblastoma, NOS (adjusted HR: 1.191, 95% CI: 1.033–1.372, *p* = 0.016).

### 3.4. Multivariable Cox Regression for Cancer-Specific Survival

Similar findings were observed for cancer-specific survival. In the multivariable Cox regression model, diagnosis during the COVID period was independently associated with worse cancer-specific survival compared with the pre-COVID period (adjusted HR: 1.048, 95% CI: 1.003–1.095, *p* = 0.035) (Table 3). Increasing age was again strongly associated with poorer CSS, with patients aged ≥80 years having more than three-fold higher cancer-specific mortality compared with those aged <50 years (adjusted HR: 3.144, 95% CI: 2.825–3.499, *p* < 0.001). Male sex was also associated with poorer CSS (adjusted HR: 1.130, 95% CI: 1.081–1.182, *p* < 0.001). Cancer-directed treatment was consistently associated with improved cancer-specific survival. Compared with no surgery, radical/total resection was associated with substantially lower cancer-specific mortality (adjusted HR: 0.573, 95% CI: 0.535–0.614, *p* < 0.001), as were subtotal resection, local excision/biopsy, extended resection, and surgery NOS/other. Receipt of radiotherapy (adjusted HR: 0.576, 95% CI: 0.532–0.623, *p* < 0.001) and chemotherapy (adjusted HR: 0.520, 95% CI: 0.482–0.561, *p* < 0.001) were also independently associated with improved CSS. Gliosarcoma was associated with poorer CSS compared with glioblastoma, NOS (adjusted HR: 1.197, 95% CI: 1.034–1.386, *p* = 0.016). Rural-urban status was not significantly associated with either OS or CSS.

### 3.5. Sensitivity and Competing-Risk Analyses

To address potential concerns regarding post-diagnosis treatment adjustment, temporal heterogeneity within the pandemic period, and competing mortality, additional sensitivity analyses were performed (Table 4). When surgery, radiotherapy, and chemotherapy were excluded from the multivariable Cox models, diagnosis during the COVID period remained modestly associated with poorer OS (HR 1.053, 95% CI 1.010–1.099, *p* = 0.016) and CSS (HR 1.052, 95% CI 1.007–1.099, *p* = 0.023). In year-specific analyses using the pre-COVID period as the reference, diagnosis in 2020 was associated with poorer OS (HR 1.064, 95% CI 1.011–1.119, *p* = 0.017) and CSS (HR 1.057, 95% CI 1.002–1.114, *p* = 0.040), whereas diagnosis in 2021 was not significantly associated with OS (HR 1.042, 95% CI 0.989–1.098, *p* = 0.122) or CSS (HR 1.047, 95% CI 0.992–1.105, *p* = 0.096). In the Fine–Gray competing-risk model, with deaths from causes other than glioblastoma treated as competing events, COVID-period diagnosis was not associated with the subdistribution hazard of cancer-specific death (SHR 0.997, 95% CI 0.955–1.042, *p* = 0.903).

### 3.6. Machine Learning-Based Prediction of 12-Month All-Cause Mortality

The performance results of the machine learning models before the COVID-19 pandemic are presented in Table 5. In this binary classification task, performance metrics were calculated separately for the “Alive” and “Dead” classes using a one-vs-rest approach, where each class was treated as the positive class while the other class was considered negative. The overall values represent the average performance across both classes. Among the evaluated models, Gradient Boosting achieved the highest overall ROC Area (0.7824) and overall accuracy (0.7002), indicating a stronger ability to discriminate between patients who survived and those who died within 12 months. Logistic Regression yielded the highest sensitivity for the “Alive” class (0.8575), correctly identifying a larger proportion of surviving patients; however, this was accompanied by a comparatively lower specificity (0.5421), reflecting a reduced ability to correctly identify patients in the opposite class. Random Forest and Support Vector Machine (SVM) produced more balanced classification results, both achieving overall accuracies of approximately 0.70. Across all models, the Matthews Correlation Coefficient (MCC) ranged from 0.40 to 0.42, suggesting a moderate positive association between the observed outcomes and the model predictions in the pre-pandemic dataset.

As shown in Table 6, the predictive performance of the models exhibited a general decline during the COVID-19 pandemic. Overall accuracy decreased across all methods, with Gradient Boosting again performing slightly better than the others at 0.6762, followed closely by Random Forest (0.6683) and SVM (0.6673). Logistic Regression showed the most pronounced difficulty in handling class imbalance during this period, maintaining relatively high sensitivity for the “Alive” class (0.8239) but demonstrating reduced ability to correctly identify the “Dead” class, as reflected in a lower specificity value (0.5191). The ROC Area values also declined compared to the pre-pandemic period, with the highest value reaching 0.7459 for Gradient Boosting. This downward trend is further supported by the MCC values, which decreased to a range of 0.33–0.35, indicating reduced overall classification robustness. In this context, sensitivity, specificity, precision, recall, and F1-score are reported separately for the “Alive” and “Dead” classes, while overall metrics are computed using macro-averaging across classes. This ensures balanced evaluation across both outcomes, particularly under conditions of class imbalance and increased data variability during the pandemic period.

Table 7 summarizes the aggregated performance across both the pre-pandemic and pandemic periods, providing an overall assessment of model robustness under varying data conditions. When trained on the combined dataset, the models exhibited a more consistent performance profile, with overall accuracies converging around 0.69. Logistic Regression achieved the highest ROC Area (0.7717) and the highest MCC (0.3870), closely followed by Gradient Boosting (0.7700). Among the evaluated methods, SVM demonstrated the most balanced F1-scores across classes, with values of 0.6663 for “Alive” and 0.7138 for “Dead,” indicating a relatively stable trade-off between precision and recall. Overall, the findings in Table 7 suggest that although the COVID-19 period introduced additional noise and variability into the dataset, the use of a larger and more heterogeneous longitudinal dataset helped preserve model performance, enabling classification results that remain close to pre-pandemic levels.

**Figure 4:**
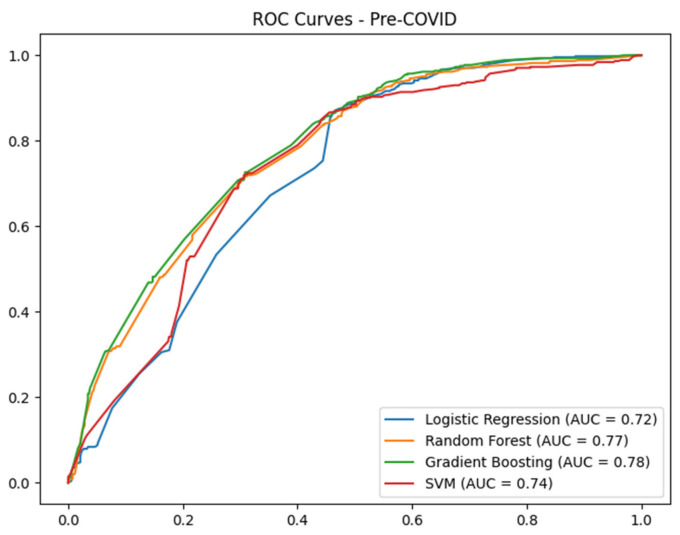
ROC curves and AUC scores for the pre-COVID-19 period.

Figure 4 illustrates the Receiver Operating Characteristic (ROC) curves and corresponding Area Under the Curve (AUC) values for the four machine learning models evaluated in the pre-pandemic cohort. Gradient Boosting achieved the highest AUC (0.78), followed closely by Random Forest (0.77). These ensemble-based models demonstrated relatively stronger discrimination, as reflected by their higher true positive rates across a range of false positive rates. Support Vector Machine (AUC = 0.74) and Logistic Regression (AUC = 0.72) showed comparatively lower but still consistent performance. The overall proximity of the ROC curves suggests that all evaluated models captured similar predictive signals from the underlying registry variables within this cohort, despite differences in algorithmic structure.

**Figure 5:**
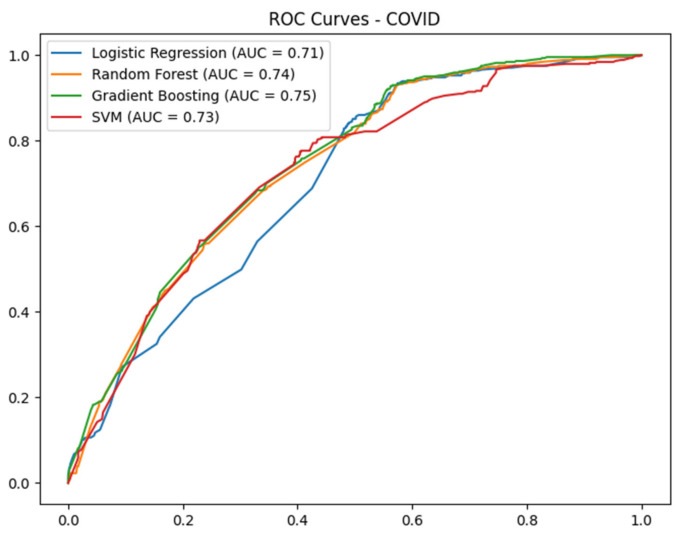
ROC curves and AUC scores for the COVID-19 period.

The ROC performance for the COVID-19 period is presented in Figure 5. The relative ranking of models remained broadly consistent, with Gradient Boosting (AUC = 0.75) and Random Forest (AUC = 0.74) continuing to show the highest discrimination, followed by SVM (AUC = 0.73) and Logistic Regression (AUC = 0.71). Compared with the pre-pandemic cohort, the AUC values show a slight reduction across models; however, no formal statistical tests were conducted to assess whether these differences are significant. The ROC curves also appear more variable, which may reflect changes in data distribution, increased heterogeneity, or reduced stability in the underlying cohort characteristics during this period, rather than a definitive change in model capability.

**Figure 6:**
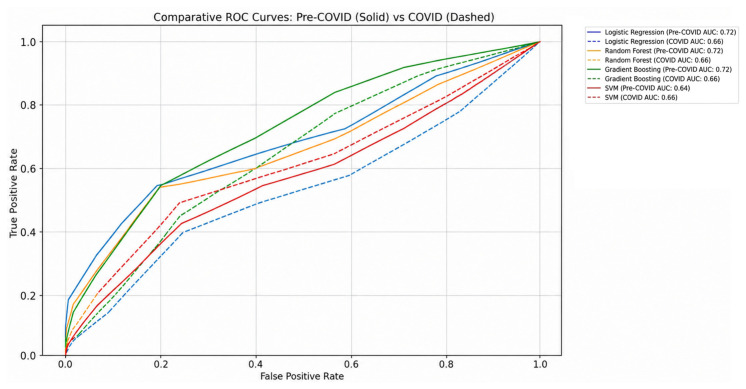
Comparative ROC curves and AUC scores before and during the COVID-19 pandemic.

Figure 6 provides an overlay of ROC curves from the pre-COVID (solid lines) and COVID-19 (dashed lines) cohorts, allowing a visual comparison of model performance across periods. Across most models, the COVID-period curves tend to lie slightly below their pre-pandemic counterparts, particularly in mid-range false positive rate regions. This pattern indicates modest differences in sensitivity–specificity trade-offs between the two cohorts; however, these visual differences should be interpreted descriptively, as no statistical comparison of ROC curves (e.g., DeLong’s test or confidence interval analysis) was performed. Therefore, the observed separation reflects potential variation in cohort characteristics and data structure rather than a confirmed statistically significant performance change.

**Figure 7:**
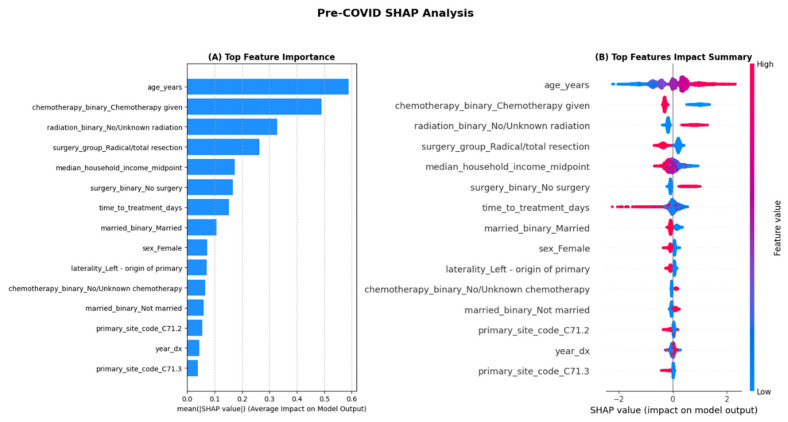
SHAP analysis for the pre-COVID-19 period.

Figure 7 presents the SHAP analysis for the pre-COVID-19 dataset, illustrating the features that contributed most to model predictions of 12-month mortality. In the global importance plot (A), age and receipt of chemotherapy emerged as the two features with the highest mean absolute SHAP values, indicating that these variables exerted the greatest influence on model output across the pre-pandemic cohort. The impact summary plot (B) further characterizes the direction of these associations: higher age values (indicated by red points) were associated with positive SHAP values, meaning that older age shifted the model’s prediction toward the “Dead” outcome. Receipt of chemotherapy (blue points) was associated with negative SHAP values, indicating that its presence shifted predicted risk toward survival. Radiation status and extent of surgical resection also contributed substantially to model predictions, with complete surgical resection associated with lower predicted mortality risk in the model output.

It should be noted that these associations reflect patterns learned from registry-based observational data and should not be interpreted as evidence of causal relationships. In particular, treatment-related features such as chemotherapy, radiation, and surgery are strong markers of patient selection in administrative datasets, and patients who received these interventions are systematically more likely to have had a favorable performance status, lower comorbidity burden, and greater access to specialized care, all of which independently influence survival. The SHAP values, therefore, capture the combined prognostic signal of treatment receipt and its associated confounders, rather than isolating any direct therapeutic effect. These findings are best understood as associative contributions to model predictions within this observational cohort.

**Figure 8:**
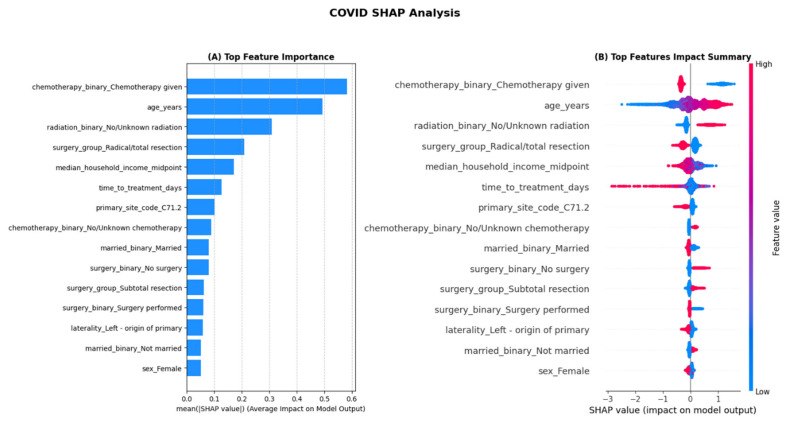
SHAP analysis for the COVID-19 period.

The SHAP analysis for the pandemic period, shown in Figure 8, reveals a shift in the relative ranking of predictor contributions compared to the pre-pandemic model. While age and chemotherapy remained the two dominant features, chemotherapy exhibited a higher mean absolute SHAP value than age during this period, indicating that the model assigned greater weight to chemotherapy receipt when generating predictions in the pandemic cohort. The impact summary (B) shows that the direction of the association between chemotherapy and predicted mortality remained consistent; receipt of chemotherapy was associated with lower predicted mortality risk, but its relative contribution to model output increased compared to the baseline period. It should be noted that this shift reflects a change in model behavior rather than a change in the underlying biological or clinical mechanism; chemotherapy receipt in registry data is strongly confounded by patient fitness, tumor characteristics, performance status, and access to care, all of which independently influence survival. The wider spread of SHAP values for treatment-related features such as surgery status and time to treatment during the pandemic period suggests greater heterogeneity in the model’s predictions across patients, consistent with increased variability in clinical trajectories during this era. These observations should be interpreted as associative patterns learned by the model from observational data, not as evidence of altered treatment efficacy or differential causal impact during the pandemic.

**Figure 9:**
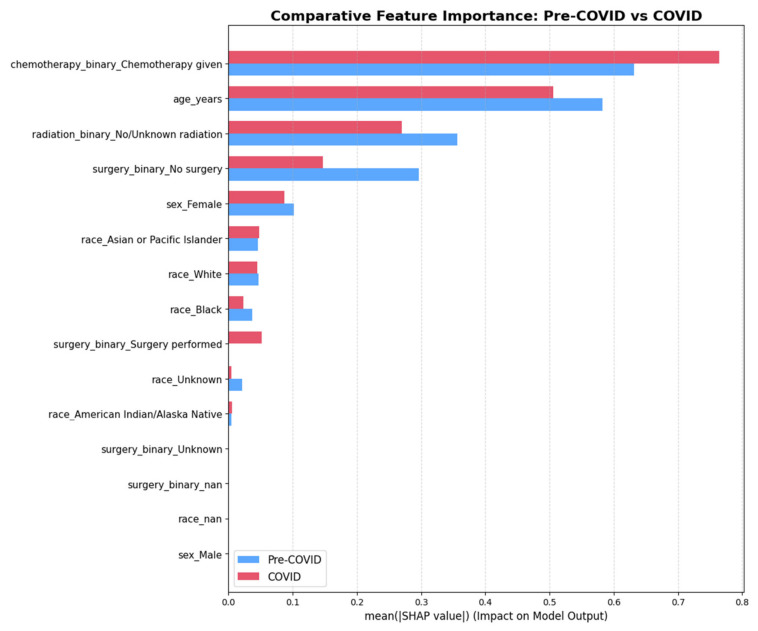
Comparative SHAP feature importance analysis before and during the COVID-19 pandemic.

Figure 9 presents a direct comparison of mean absolute SHAP values between the pre-COVID-19 (blue bars) and COVID-19 (red bars) periods. Chemotherapy showed the largest increase in model-assigned feature importance across the two periods, while age and radiation therapy exhibited modest decreases in relative contribution. Surgery-related variables and demographic features, including sex and race, showed broadly stable importance rankings across both periods, with only minor fluctuations. These cross-period differences in feature importance reflect changes in the statistical associations captured by the model within each temporal dataset and should not be interpreted as evidence that chemotherapy became biologically or causally more important during the pandemic. In registry-based observational data, treatment receipt is a complex composite marker that encodes patient selection, clinical eligibility, institutional practice patterns, and access to care, all of which may have shifted during the pandemic in ways that are not separable from treatment effects in the model. The apparent increase in the predictive weight of chemotherapy may therefore reflect pandemic-related changes in which patients received treatment, and thus which patients survived, rather than any change in the pharmacological benefit of the treatment itself. Accordingly, these findings should be understood as cross-temporal differences in associative model behavior rather than evidence of shifting clinical or biological mechanisms.

## 4. Discussion

In this population-based SEER study of adult patients with glioblastoma, diagnosis during the COVID-19 period was associated with slightly poorer overall survival and cancer-specific survival compared with diagnosis during the pre-COVID period. This association remained statistically significant after adjustment for demographic, tumor-related, socioeconomic, and treatment-related variables. However, the magnitude of the association was modest, with adjusted hazard ratios close to 1.05 for both outcomes. Therefore, these findings should not be interpreted as evidence of a large pandemic-related survival effect, but rather as a small yet measurable population-level survival difference among patients diagnosed during the pandemic period. The additional sensitivity analyses further support a cautious interpretation of the survival findings. When surgery, radiotherapy, and chemotherapy were excluded from the multivariable Cox models, COVID-period diagnosis remained modestly associated with poorer OS and CSS, suggesting that the observed association was not entirely dependent on adjustment for post-diagnosis treatment variables. However, the year-specific analyses indicated that the association was mainly apparent among patients diagnosed in 2020, whereas estimates for 2021 were smaller and not statistically significant. This pattern may reflect greater disruption during the early pandemic period, followed by partial adaptation of cancer care pathways, although this interpretation remains speculative because SEER does not include information on hospital-level service disruption, COVID-19 infection status, treatment delays, or neuro-oncology pathway reorganization.

The Fine-Gray competing-risk analysis also provided an important complementary perspective. In the cause-specific Cox models, COVID-period diagnosis was associated with slightly poorer CSS; however, in the Fine-Gray model treating deaths from causes other than glioblastoma as competing events, COVID-period diagnosis was not associated with the subdistribution hazard of cancer-specific death. This finding suggests that the cancer-specific survival result should be interpreted cautiously and that competing mortality, censoring structure, and registry-based cause-of-death classification may influence the observed association. Therefore, the present results are best interpreted as evidence of a modest population-level survival difference during the pandemic period rather than proof of a direct pandemic-related increase in glioblastoma-specific mortality.

Glioblastoma is an aggressive adult primary brain malignancy with limited survival despite multimodal treatment. Standard management generally requires the timely coordination of neurosurgical evaluation, maximal safe resection when feasible, radiotherapy, and temozolomide-based systemic therapy [20]. Because of this rapidly progressive clinical course, glioblastoma represents a particularly relevant tumor type for evaluating whether pandemic-related disruptions in cancer care were associated with measurable survival consequences [21]. At the same time, glioblastoma is often treated as a high-priority neuro-oncological emergency, and many centers likely attempted to preserve urgent diagnostic and treatment pathways during the pandemic. This may partly explain why the observed survival difference in the present study was statistically significant but clinically modest.

The association between COVID-period diagnosis and poorer survival may reflect the cumulative effect of several pandemic-related changes in cancer care pathways. During the pandemic, diagnostic evaluation, neurosurgical scheduling, radiotherapy planning, chemotherapy delivery, multidisciplinary decision-making, follow-up care, and supportive services may have been affected by health-system pressure, infection-control measures, resource reallocation, and patient-level barriers to hospital attendance [22]. Systematic reviews have shown that the COVID-19 pandemic disrupted multiple components of cancer care, including diagnosis, surgery, radiotherapy, systemic treatment, and follow-up services [6,7]. However, the SEER database does not provide information on COVID-19 infection status, hospital capacity, neurosurgical waiting times, treatment interruptions, or patient-level reasons for delayed or omitted treatment. Therefore, the present findings should be interpreted as an association between pandemic-period diagnosis and survival, rather than direct evidence that any specific pandemic-related mechanism caused worse outcomes.

Previous studies evaluating glioma and glioblastoma care during the COVID-19 pandemic have reported heterogeneous findings. In a Canadian cohort of patients with grade 4 glioma treated with radiotherapy, Chahal et al. reported that grade 4 gliomas are rapidly progressive tumors in which diagnostic or treatment delays may place patients at risk of poorer outcomes. Their study also observed changes in treatment patterns during the pandemic period, including differences in surgical management, although survival outcomes were not uniformly worsened [12]. Similarly, Neff et al. examined treatment patterns in glioblastoma during the COVID-19 pandemic and emphasized the need to determine whether observed treatment disruptions translated into differences in outcomes, particularly outside academic centers [13]. These studies support the rationale for evaluating glioblastoma outcomes at the population level, where smaller but broader effects may be detectable.

However, not all studies have demonstrated worse glioblastoma or high-grade glioma survival during the pandemic. Some institutional cohorts have suggested that survival remained stable, possibly because urgent neuro-oncology services were relatively protected despite wider health-care disruption. Qin et al. reported that glioblastoma survival remained broadly stable across pre-COVID and post-COVID periods, while advanced age, bilateral tumors, and passive treatment remained important prognostic factors [15]. Karamani et al. also reported no significant survival difference between pre-pandemic and pandemic periods in an institutional neuro-oncology cohort [14]. These findings are not necessarily inconsistent with the present SEER-based analysis. Single-center or surgically selected cohorts may capture patients treated in specialized systems where urgent glioblastoma pathways were maintained, whereas SEER includes a broader population, including patients who may not have undergone surgery or who may have experienced differences in access to timely multimodal care.

The present findings are also consistent with broader population-based evidence that the COVID-19 pandemic had measurable but variable effects on cancer survival. Hong et al. used SEER data and reported a decline in 1-year relative survival for all cancer sites combined during the early pandemic period, with the magnitude of decline varying by cancer type [9]. More recently, Burus et al. reported worse short-term survival among patients diagnosed with cancer in 2020 and 2021 compared with those diagnosed before the pandemic, estimating excess cancer-related deaths within 1 year of diagnosis during the first two pandemic years [8]. These studies support the concept that pandemic-related disruption did not affect all cancers equally, and that tumor-specific analyses are needed. In this context, the small but significant survival difference observed in glioblastoma may represent the combined effect of pandemic-era pressures on a malignancy that was clinically prioritized but still vulnerable to disruption.

In addition to conventional survival analyses, this study applied machine learning models to predict 12-month all-cause mortality using routinely available registry variables. The models demonstrated moderate discriminatory performance, indicating that SEER-based demographic, tumor-related, socioeconomic, and treatment-related variables contain meaningful prognostic signals for mortality risk stratification. In the pre-COVID cohort, ensemble-based methods such as Gradient Boosting and Random Forest achieved relatively higher ROC-AUC values compared to other algorithms, suggesting slightly stronger class separability within this dataset. In the COVID-period cohort, a modest reduction in ROC-AUC values was observed across models; however, no formal statistical tests were performed to evaluate whether these differences are statistically significant. Accordingly, any apparent differences in discriminative performance between periods should be interpreted cautiously and in a descriptive manner. These variations may be influenced by changes in cohort composition, sample size, follow-up structure, or temporal shifts in data distribution and model stability, rather than representing a definitive change in underlying clinical or biological predictability.

The machine learning findings are consistent with the growing literature on glioblastoma survival prediction. Poursaeed et al. summarized machine learning and deep learning approaches for glioblastoma survival prediction and emphasized that model performance depends strongly on the type and quality of input variables, including clinical, molecular, imaging, radiomic, and omics data [18]. Babaei Rikan et al. also demonstrated the feasibility of using SEER data for glioblastoma survival prediction with modern machine learning and deep learning techniques [19]. Compared with imaging or molecular-based models, the present analysis used routinely available population registry variables. This approach is pragmatic for population-level risk stratification, but it is less individualized than models incorporating molecular markers, radiological features, performance status, or detailed treatment timing.

The SHAP analyses identified age and treatment-related variables, particularly chemotherapy, radiotherapy, and surgery, as the features with the greatest contribution to model predictions of 12-month mortality across all cohorts. These associations are directionally consistent with the Cox proportional hazards models, in which increasing age was associated with poorer survival and receipt of each treatment modality was associated with improved outcomes. This concordance across modeling frameworks lends face validity to the learned associations and suggests that the machine learning models captured clinically coherent prognostic patterns.

However, these findings must be interpreted with important limitations in mind. SHAP values quantify the contribution of each feature to a model’s predictions; they do not establish causal importance, nor do they isolate the independent clinical effect of any single variable. In registry-based observational data, treatment receipt is a composite marker that is heavily confounded by factors that are often incompletely captured or entirely absent from administrative datasets. Patients who received chemotherapy, radiotherapy, or surgery are systematically different from those who did not: they are more likely to have better performance status, lower tumor burden, fewer comorbidities, greater access to specialized care, and stronger physician and patient motivation to pursue aggressive therapy. The prognostic signal attributed to treatment variables by the model, therefore, reflects this entire constellation of underlying factors, not treatment efficacy in isolation.

The observed increase in the relative model-assigned importance of chemotherapy during the pandemic period is particularly subject to this interpretive caution. Rather than indicating that chemotherapy became more clinically effective or mechanistically central during the pandemic, this shift more plausibly reflects pandemic-related changes in patient selection for treatment, disruptions to care delivery that altered which patients accessed chemotherapy, or shifts in the composition of the surviving patient population. Consequently, the contributions of treatment-related predictors in both the machine learning models and the SHAP analyses should be interpreted as prognostic associations within an observational dataset, not as estimates of causal treatment effects. Causal inference regarding treatment benefit in this population would require methodological approaches specifically designed for confounded observational data, such as propensity score methods or instrumental variable analysis, which are beyond the scope of the present study.

This study has several strengths. First, it used a large population-based SEER cohort of adult patients with glioblastoma, allowing evaluation of survival outcomes beyond single-center or regional experiences. Second, both overall survival and cancer-specific survival were analyzed, providing complementary perspectives on mortality during the pre-COVID and COVID periods [23,24]. Third, the multivariable models adjusted for multiple demographic, tumor-related, socioeconomic, and treatment-related factors. Lastly, the integration of conventional survival analysis with machine learning-based 12-month mortality prediction provides both epidemiological and predictive perspectives on glioblastoma outcomes during the pandemic period.

Several limitations should also be acknowledged. SEER does not include several clinically important glioblastoma-specific variables, including IDH mutation status, MGMT promoter methylation, Karnofsky or ECOG performance status, steroid use, neurological status, tumor volume, imaging-defined extent of resection, recurrence, or progression data. These factors are important prognostic determinants and could not be included in the present analysis. Also, treatment information in SEER is limited. The database does not provide detailed information on radiotherapy dose, fractionation, temozolomide regimen, chemotherapy completion, treatment interruption, or reasons for treatment omission. In addition, some treatment variables combine “no” and “unknown” categories, which may introduce misclassification. This study could not directly measure COVID-19 infection status, hospital-level pandemic burden, intensive care capacity, neurosurgical waiting times, patient refusal of treatment, or specific delays between symptoms, diagnosis, surgery, radiotherapy, and chemotherapy. Therefore, the observed association between COVID-period diagnosis and poorer survival cannot be attributed to a specific pandemic-related mechanism. An additional limitation concerns the interpretation of treatment variables in observational registry data. Surgery, radiotherapy, and chemotherapy are recorded as treatment receipt variables and occur after diagnosis; therefore, including them as fixed covariates in Cox models may introduce immortal-time bias, treatment-selection bias, or overadjustment if treatment lies on the pathway between diagnosis period and survival. Although we performed sensitivity analyses excluding these post-diagnosis treatment variables, residual confounding by clinical fitness, tumor burden, performance status, neurological status, access to care, physician decision-making, and patient preference remains possible. Similarly, the Fine-Gray competing-risk analysis should be interpreted as a sensitivity analysis, because SEER cause-specific death classification may be subject to misclassification and does not capture detailed clinical circumstances surrounding death.

The findings suggest that adult glioblastoma patients diagnosed during the COVID-19 period experienced a small but measurable survival disadvantage at the population level. This does not imply that glioblastoma care was broadly disrupted in all settings or that the pandemic produced a large deterioration in glioblastoma outcomes. Rather, the results suggest that even highly prioritized malignancies may be vulnerable to subtle survival effects during periods of health-system stress [25,26]. Future studies should incorporate molecular markers, performance status, detailed treatment timing, COVID-19 infection status, hospital-level factors, and neuro-oncology service capacity to clarify the mechanisms underlying these differences. In addition, externally validated prediction models combining registry data with clinical, molecular, imaging, and treatment-timing variables may improve short-term mortality prediction in glioblastoma.

## 5. Conclusions

In conclusion, adult glioblastoma patients diagnosed during the COVID-19 period had slightly poorer overall and cancer-specific survival in conventional survival analyses compared with those diagnosed before the pandemic. Although the magnitude of this association was modest, the difference remained statistically significant after multivariable adjustment and in sensitivity Cox models excluding post-diagnosis treatment variables. However, competing-risk analysis did not show a significant association between COVID-period diagnosis and the subdistribution hazard of cancer-specific death, supporting cautious interpretation of the cancer-specific survival findings. Machine learning models based on routinely available registry variables showed moderate ability to predict 12-month all-cause mortality, with slightly lower performance during the COVID period. These findings suggest that pandemic-era conditions may have had measurable but limited population-level consequences for glioblastoma outcomes and highlight the need for further studies using more detailed clinical, molecular, imaging, and treatment-timing data.

## Figures and Tables

**Table 1:** Baseline characteristics of glioblastoma patients diagnosed before and during the COVID-19 pandemic.

Variable	Overall n (%)	Pre-COVID n (%)	COVID n (%)	*p*
Total	9914	4819	5095	
Age group				0.034
<50	1167 (11.8)	580 (12.0)	587 (11.5)	
50–59	2233 (22.5)	1135 (23.6)	1098 (21.6)	
60–69	3274 (33.0)	1595 (33.1)	1679 (33.0)	
70–79	2541 (25.6)	1181 (24.5)	1360 (26.7)	
≥80	699 (7.1)	328 (6.8)	371 (7.3)	
Sex				0.786
Female	4083 (41.2)	1978 (41.1)	2105 (41.3)	
Male	5831 (58.8)	2841 (59.0)	2990 (58.7)	
Race				0.841
White	8568 (86.4)	4174 (86.6)	4394 (86.2)	
Black	610 (6.2)	294 (6.1)	316 (6.2)	
Asian/Pacific Islander	610 (6.2)	287 (6.0)	323 (6.3)	
Other/Unknown	126 (1.3)	64 (1.3)	62 (1.2)	
Marital status				0.135
Married	6306 (63.6)	3064 (63.6)	3242 (63.6)	
Not married	3309 (33.4)	1593 (33.1)	1716 (33.7)	
Unknown	299 (3.0)	162 (3.4)	137 (2.7)	
Tumor site				0.259
Frontal lobe	3002 (30.3)	1427 (29.6)	1575 (30.9)	
Temporal lobe	2619 (26.4)	1258 (26.1)	1361 (26.7)	
Parietal lobe	1550 (15.6)	751 (15.6)	799 (15.7)	
Other/NOS	2743 (27.7)	1383 (28.7)	1360 (26.7)	
Histology				0.411
Glioblastoma, NOS	9628 (97.1)	4670 (96.9)	4958 (97.3)	
GBM variants	286 (2.9)	149 (3.1)	137 (2.7)	
Surgery				0.018
No surgery	1571 (15.9)	779 (16.2)	792 (15.5)	
Biopsy/local excision	1373 (13.9)	699 (14.5)	674 (13.2)	
Subtotal resection	2794 (28.2)	1393 (28.9)	1401 (27.5)	
Radical/total/extended resection	3814 (38.5)	1797 (37.3)	2017 (39.6)	
Other/unknown surgery	362 (3.7)	151 (3.1)	211 (4.1)	
Radiotherapy				0.038
Radiation given	7369 (74.3)	3627 (75.3)	3742 (73.4)	
No/unknown radiation	2545 (25.7)	1192 (24.7)	1353 (26.6)	
Chemotherapy				0.087
Chemotherapy given	7047 (71.1)	3464 (71.9)	3583 (70.3)	
No/unknown chemotherapy	2867 (28.9)	1355 (28.1)	1512 (29.7)	
Rural-urban status				0.640
Metropolitan	8786 (88.6)	4271 (88.6)	4515 (88.6)	
Nonmetropolitan/unknown	1128 (11.4)	548 (11.4)	580 (11.4)	

Values are presented as n (%). Categorical variables were compared using Pearson’s chi-square test. GBM variants include giant cell glioblastoma and gliosarcoma. Pre-COVID period was defined as 2018–2019 and COVID period as 2020–2021.

**Table 2:** Multivariable Cox regression analysis for overall survival among glioblastoma patients diagnosed before and during the COVID-19 pandemic.

Variable	Category	Adjusted HR	95% CI	*p*-Value
Diagnosis period	Pre-COVID	Reference		
	COVID	1.050	1.006–1.095	0.025
Age group	<50	Reference		
	50–59	1.389	1.283–1.505	<0.001
	60–69	1.767	1.638–1.906	<0.001
	70–79	2.364	2.185–2.558	<0.001
	≥80	3.123	2.817–3.463	<0.001
Sex	Female	Reference		
	Male	1.130	1.083–1.180	<0.001
Race	White	Reference		
	American Indian/Alaska Native	0.867	0.657–1.142	0.309
	Asian or Pacific Islander	0.824	0.753–0.902	<0.001
	Black	0.912	0.835–0.997	0.042
	Unknown	0.410	0.300–0.561	<0.001
Marital status	Not married	Reference		
	Married	0.905	0.865–0.948	<0.001
	Unknown	0.855	0.752–0.972	0.016
Histology	Glioblastoma, NOS	Reference		
	Giant cell glioblastoma	0.828	0.632–1.086	0.173
	Gliosarcoma	1.191	1.033–1.372	0.016
Surgery	No surgery	Reference		
	Local excision/biopsy	0.767	0.710–0.829	<0.001
	Subtotal resection	0.767	0.717–0.821	<0.001
	Radical/total resection	0.581	0.544–0.621	<0.001
	Extended resection	0.696	0.600–0.807	<0.001
	Surgery, NOS/other	0.592	0.517–0.678	<0.001
Radiotherapy	No/unknown radiation	Reference		
	Radiation given	0.560	0.519–0.605	<0.001
Chemotherapy	No/unknown chemotherapy	Reference		
	Chemotherapy given	0.525	0.487–0.564	<0.001
Rural-urban status	Metropolitan	Reference		
	Nonmetropolitan	1.027	0.953–1.107	0.478

HR, hazard ratio; CI, confidence interval. Overall survival was defined as time from diagnosis to death from any cause. The multivariable Cox model was adjusted for diagnosis period, age group, sex, race, marital status, primary tumor site, histology, laterality, surgery, radiotherapy, chemotherapy, rural-urban status, and median household income.

**Table 3:** Multivariable Cox regression analysis for cancer-specific survival among glioblastoma patients diagnosed before and during the COVID-19 pandemic.

Variable	Category	Adjusted HR	95% CI	*p*-Value
Diagnosis period	Pre-COVID	Reference		
	COVID	1.048	1.003–1.095	0.035
Age group	<50	Reference		
	50–59	1.395	1.285–1.515	<0.001
	60–69	1.762	1.629–1.906	<0.001
	70–79	2.348	2.164–2.548	<0.001
	≥80	3.144	2.825–3.499	<0.001
Sex	Female	Reference		
	Male	1.130	1.081–1.182	<0.001
Race	White	Reference		
	American Indian/Alaska Native	0.853	0.640–1.138	0.280
	Asian or Pacific Islander	0.805	0.733–0.885	<0.001
	Black	0.870	0.792–0.955	0.004
	Unknown	0.389	0.279–0.543	<0.001
Marital status	Not married	Reference		
	Married	0.917	0.875–0.962	<0.001
	Unknown	0.832	0.726–0.953	0.008
Histology	Glioblastoma, NOS	Reference		
	Giant cell glioblastoma	0.835	0.632–1.104	0.207
	Gliosarcoma	1.197	1.034–1.386	0.016
Surgery	No surgery	Reference		
	Local excision/biopsy	0.765	0.706–0.829	<0.001
	Subtotal resection	0.754	0.703–0.809	<0.001
	Radical/total resection	0.573	0.535–0.614	<0.001
	Extended resection	0.695	0.596–0.810	<0.001
	Surgery, NOS/other	0.580	0.504–0.667	<0.001
Radiotherapy	No/unknown radiation	Reference		
	Radiation given	0.576	0.532–0.623	<0.001
Chemotherapy	No/unknown chemotherapy	Reference		
	Chemotherapy given	0.520	0.482–0.561	<0.001
Rural-urban status	Metropolitan	Reference		
	Nonmetropolitan	1.024	0.948–1.107	0.543

HR, hazard ratio; CI, confidence interval. Cancer-specific survival was defined using the SEER cause-specific death classification. Deaths from other causes were censored. The multivariable Cox model was adjusted for diagnosis period, age group, sex, race, marital status, primary tumor site, histology, laterality, surgery, radiotherapy, chemotherapy, rural-urban status, and median household income.

**Table 4:** Sensitivity and competing-risk analyses for survival outcomes among glioblastoma patients diagnosed before and during the COVID-19 pandemic.

Analysis	Outcome	Model/Comparison	Estimate	95% CI	*p*-Value
Main Cox model	OS	COVID vs. pre-COVID, treatment-adjusted	HR 1.050	1.006–1.095	0.025
Main Cox model	CSS	COVID vs. pre-COVID, treatment-adjusted	HR 1.048	1.003–1.095	0.035
Sensitivity Cox model	OS	Excluding surgery, radiotherapy, and chemotherapy	HR 1.053	1.010–1.099	0.016
Sensitivity Cox model	CSS	Excluding surgery, radiotherapy, and chemotherapy	HR 1.052	1.007–1.099	0.023
Year-specific sensitivity analysis	OS	2020 vs. 2018–2019	HR 1.064	1.011–1.119	0.017
Year-specific sensitivity analysis	OS	2021 vs. 2018–2019	HR 1.042	0.989–1.098	0.122
Year-specific sensitivity analysis	CSS	2020 vs. 2018–2019	HR 1.057	1.002–1.114	0.040
Year-specific sensitivity analysis	CSS	2021 vs. 2018–2019	HR 1.047	0.992–1.105	0.096
Fine–Gray competing-risk model	CSS	COVID vs. pre-COVID; other-cause death as competing event	SHR 0.997	0.955–1.042	0.903

OS, overall survival; CSS, cancer-specific survival; HR, hazard ratio; SHR, subdistribution hazard ratio; CI, confidence interval. Main Cox models were adjusted for diagnosis period, age group, sex, race, marital status, primary tumor site, histology, laterality, surgery, radiotherapy, chemotherapy, rural-urban status, and median household income. Sensitivity Cox models excluded post-diagnosis treatment variables, including surgery, radiotherapy, and chemotherapy. The Fine–Gray model treated death from causes other than glioblastoma as a competing event.

**Table 5:** Performance results of Machine Learning methods before the COVID-19 pandemic.

Methods		Performance Metrics							
		Accuracy	Sensitivity	Specificity	Precision	Recall	F1-Score	ROC Area	MCC
Logistic Regression	Alive	0.8575	0.8575	0.8575	0.6133	0.8575	0.7151	0.7219	0.4151
	Dead	0.5421	0.5421	0.5421	0.8179	0.5421	0.6521	0.7219	0.4151
	Overall	0.6867	0.8575	0.5421	0.7241	0.6867	0.6810	0.7219	0.4151
Random Forest	Alive	0.7195	0.7195	0.7195	0.6570	0.7195	0.6868	0.7695	0.4001
	Dead	0.6820	0.6820	0.6820	0.7417	0.6820	0.7106	0.7695	0.4001
	Overall	0.6992	0.7195	0.6820	0.7029	0.6992	0.6997	0.7695	0.4001
Gradient Boosting	Alive	0.7081	0.7081	0.7081	0.6617	0.7081	0.6842	0.7824	0.4003
	Dead	0.6935	0.6935	0.6935	0.7373	0.6935	0.7147	0.7824	0.4003
	Overall	0.7002	0.7081	0.6935	0.7026	0.7002	0.7007	0.7824	0.4003
Support Vector Machine	Alive	0.7262	0.7262	0.7262	0.6551	0.7262	0.6888	0.7379	0.4012
	Dead	0.6762	0.6762	0.6762	0.7447	0.6762	0.7008	0.7379	0.4012
	Overall	0.6992	0.7262	0.6762	0.7036	0.6992	0.6997	0.7379	0.4012

MCC: Matthews correlation coefficient, ROC: Receiver Operating Characteristic.

**Table 6:** Performance results of Machine Learning methods during the COVID-19 pandemic.

Methods		Performance Metrics							
		Accuracy	Sensitivity	Specificity	Precision	Recall	F1-Score	ROC Area	MCC
Logistic Regression	Alive	0.8239	0.8239	0.8239	0.5685	0.8239	0.6728	0.7005	0.3522
	Dead	0.5191	0.5191	0.5191	0.7931	0.5191	0.6275	0.7005	0.3522
	Overall	0.6516	0.8239	0.5191	0.6955	0.6516	0.6472	0.7005	0.3522
Random Forest	Alive	0.6862	0.6862	0.6862	0.6044	0.6862	0.6427	0.7384	0.3379
	Dead	0.6545	0.6545	0.6545	0.7306	0.6545	0.6905	0.7384	0.3379
	Overall	0.6683	0.6862	0.6545	0.6757	0.6683	0.6697	0.7384	0.3379
Gradient Boosting	Alive	0.6840	0.6840	0.6840	0.6146	0.6840	0.6474	0.7459	0.3513
	Dead	0.6701	0.6701	0.6701	0.7338	0.6701	0.7005	0.7459	0.3513
	Overall	0.6762	0.6840	0.6701	0.6820	0.6762	0.6775	0.7459	0.3513
Support Vector Machine	Alive	0.7494	0.7494	0.7494	0.5929	0.7494	0.6620	0.7266	0.3523
	Dead	0.6042	0.6042	0.6042	0.7582	0.6042	0.6725	0.7266	0.3523
	Overall	0.6673	0.7494	0.6042	0.6863	0.6673	0.6679	0.7266	0.3523

MCC: Matthews correlation coefficient, ROC: Receiver Operating Characteristic.

**Table 7:** Performance results of Machine Learning methods before and during the COVID-19 pandemic.

Methods		Performance Metrics							
		Accuracy	Sensitivity	Specificity	Precision	Recall	F1-Score	ROC Area	MCC
Logistic Regression	Alive	0.7121	0.7121	0.7121	0.6311	0.7121	0.6692	0.7717	0.3870
	Dead	0.6780	0.6780	0.6780	0.7527	0.6780	0.7134	0.7717	0.3870
	Overall	0.6929	0.7121	0.7121	0.6311	0.7121	0.6692	0.7717	0.3870
Random Forest	Alive	0.7064	0.7064	0.7064	0.6273	0.7064	0.6645	0.7632	0.3786
	Dead	0.6753	0.6753	0.6753	0.7483	0.6753	0.7099	0.7632	0.3786
	Overall	0.6889	0.7064	0.6753	0.6955	0.6889	0.6901	0.7632	0.3786
Gradient Boosting	Alive	0.7121	0.7121	0.7121	0.6279	0.7121	0.6674	0.7700	0.3825
	Dead	0.6735	0.6735	0.6735	0.7515	0.6735	0.7104	0.7700	0.3825
	Overall	0.6904	0.7121	0.6735	0.6976	0.6904	0.6916	0.7700	0.3825
Support Vector Machine	Alive	0.7052	0.7052	0.7052	0.6315	0.7052	0.6663	0.7027	0.3837
	Dead	0.6816	0.6816	0.6816	0.7493	0.6816	0.7138	0.7027	0.3837
	Overall	0.6919	0.7052	0.6816	0.6979	0.6919	0.6931	0.7027	0.3837

MCC: Matthews correlation coefficient, ROC: Receiver Operating Characteristic.

## Data Availability

The data used in this study are publicly available from the Surveillance, Epidemiology, and End Results (SEER) Program of the National Cancer Institute upon completion of the SEER Research Data Agreement. Data were extracted using SEER*Stat software version 9.0.42.2 from the “Incidence-SEER Research Data, 17 Registries, November 2024 Submission, released April 2025” database. Data access was granted under SEER Research Data Agreement number SAR0115042. No new data were created in this study.

## Supplementary Materials

The following supporting information can be downloaded at: https://github.com/omerecinar/MDPI-Medicina-Manuscript-Colab-Codes.git (accessed on 5 May 2026). Python source codes and execution scripts for the reported ML models and SHAP analysis.

## Abbreviations

The following abbreviations are used in this manuscript:

AUC	Area Under the Curve
CI	Confidence Interval
COVID-19	Coronavirus Disease 2019
CSS	Cancer-Specific Survival
GBM	Glioblastoma
HR	Hazard Ratio
ICD-O-3	International Classification of Diseases for Oncology, Third Edition
MCC	Matthews Correlation Coefficient
ML	Machine Learning
NCI	National Cancer Institute
NOS	Not Otherwise Specified
OS	Overall Survival
ROC	Receiver Operating Characteristic
SEER	Surveillance, Epidemiology, and End Results
SHAP	Shapley Additive Explanations
SVM	Support Vector Machine

## References

[1] Singh S., Dey D., Barik D., Mohapatra I., Kim S., Sharma M., Prasad S., Wang P., Singh A., Singh G. (2025). Glioblastoma at the crossroads: Current understanding and future therapeutic horizons. Signal Transduct. Target. Ther..

[2] Louis D.N., Perry A., Wesseling P., Brat D.J., Cree I.A., Figarella-Branger D., Hawkins C., Ng H.K., Pfister S.M., Reifenberger G. (2021). The 2021 WHO Classification of Tumors of the Central Nervous System: A summary. Neuro-Oncology.

[3] Siegel R.L., Kratzer T.B., Giaquinto A.N., Sung H., Jemal A. (2025). Cancer statistics, 2025. CA Cancer J. Clin..

[4] Geens W., Rizani G., Del Gaudio N., Buyck F., Van Gestel F., Bruneau M., Neyns B., Duerinck J. (2025). Extent of resection and its association with overall survival in newly diagnosed IDH wildtype glioblastoma treated with concomitant radiochemotherapy: A systematic review and meta-analysis. Brain Spine.

[5] Valerius A.R., Webb L.M., Thomsen A., Lehrer E.J., Breen W.G., Campian J.L., Riviere-Cazaux C., Burns T.C., Sener U. (2024). Review of Novel Surgical, Radiation, and Systemic Therapies and Clinical Trials in Glioblastoma. Int. J. Mol. Sci..

[6] Riera R., Bagattini Â.M., Pacheco R.L., Pachito D.V., Roitberg F., Ilbawi A. (2021). Delays and Disruptions in Cancer Health Care Due to COVID-19 Pandemic: Systematic Review. JCO Glob. Oncol..

[7] Shah R., Hanna N.M., Loo C.E., David M., Mafra A., Fink H., McFerran E., Garcia M., Ghodssighassemabadi R., Acharya S. (2025). The global impact of the COVID-19 pandemic on delays and disruptions in cancer care services: A systematic review and meta-analysis. Nat. Cancer.

[8] Burus T., Damgacioglu H., Huang B., Tucker T.C., Deshmukh A.A., Lang Kuhs K.A. (2026). Survival of Patients Diagnosed with Cancer During the COVID-19 Pandemic. JAMA Oncol..

[9] Hong Y.D., Howlader N., Noone A.M., Mariotto A.B. (2025). Assessing the effect of the COVID-19 pandemic on 1-year cancer survival in the United States. J. Natl. Cancer Inst..

[10] Azab M.A., Azzam A.Y. (2021). Impact of COVID-19 pandemic on the management of glioma patients around the world. An evidence-based review. Brain Disord..

[11] Mischkulnig M., Hopp B., Wadiura L.I., Khalaveh F., Kiesel B., Rössler K., Widhalm G., Dorfer C. (2023). Treatment of high-grade glioma patients during the COVID-19 pandemic: Impact on overall survival, tumor size and delay of treatment. PLoS ONE.

[12] Chahal M., Aljawi G., Harrison R., Nichol A., Thiessen B. (2023). Treatment Patterns and Outcomes of Patients with Grade 4 Glioma Treated with Radiation during the COVID-19 Pandemic. Curr. Oncol..

[13] Neff C., Price M., Cioffi G., Waite K.A., Kruchko C., Iorgulescu J.B., Barnholtz-Sloan J.S., Ostrom Q.T. (2024). The impact of the COVID-19 pandemic on treatment patterns in glioblastoma. Neuro-Oncology.

[14] Karamani L., McLean A.L., Kamp M.A., Mayer T.E., Müller W., Dinc N., Senft C. (2023). Tumor size, treatment patterns, and survival in neuro-oncology patients before and during the COVID-19 pandemic. Neurosurg. Rev..

[15] Qin L., Li H., Zheng D., Lin S., Ren X. (2024). Glioblastoma patients’ survival and its relevant risk factors during the pre-COVID-19 and post-COVID-19 pandemic: Real-world cohort study in the USA and China. Int. J. Surg..

[16] Tang Y., Lin L., Han Z., Yi X., Li X., Meng L., Zhu Y., Ai J. (2025). Impact of the COVID-19 pandemic in 2020 on the diagnosis, treatment, and prognosis of major cancers. Int. J. Surg..

[17] Duman A., Sun X., Thomas S., Powell J.R., Spezi E. (2024). Reproducible and Interpretable Machine Learning-Based Radiomic Analysis for Overall Survival Prediction in Glioblastoma Multiforme. Cancers.

[18] Poursaeed R., Mohammadzadeh M., Safaei A.A. (2024). Survival prediction of glioblastoma patients using machine learning and deep learning: A systematic review. BMC Cancer.

[19] Babaei Rikan S., Sorayaie Azar A., Naemi A., Bagherzadeh Mohasefi J., Pirnejad H., Wiil U.K. (2024). Survival prediction of glioblastoma patients using modern deep learning and machine learning techniques. Sci. Rep..

[20] Sipos D., Raposa B.L., Freihat O., Simon M., Mekis N., Cornacchione P., Kovács Á. (2025). Glioblastoma: Clinical Presentation, Multidisciplinary Management, and Long-Term Outcomes. Cancers.

[21] Chakravarti S., Gupta S.R., Myneni S., Elshareif M., Rogers J.L., Caraway C., Ahmed A.K., Schreck K.C., Kamson D.O., Holdhoff M. (2025). Clinical Outcome Assessment Tools for Evaluating the Management of Gliomas. Cancers.

[22] Mc Fadden S., Flood T., Shepherd P., Gilleece T. (2022). Impact of COVID-19 on service delivery in radiology and radiotherapy. Radiography.

[23] Redfors B. (2023). Improving Registry-Based Observational Comparative Effectiveness Studies by Prospectively Incorporating Robust Treatment Preference Instruments. JACC Adv..

[24] Gallicchio L., Elena J.W., Fagan S., Carter M., Hamilton A.S., Hastert T.A., Hunter L.L., Li J., Lynch C.F., Milam J. (2020). Utilizing SEER Cancer Registries for Population-Based Cancer Survivor Epidemiologic Studies: A Feasibility Study. Cancer Epidemiol. Biomark. Prev..

[25] Claveau S., Mahmood F., Amir B., Kwan J.J.W., White C., Vipond J., Iannattone L. (2024). COVID-19 and Cancer Care: A Review and Practical Guide to Caring for Cancer Patients in the Era of COVID-19. Curr. Oncol..

[26] Attieh S., Loiselle C.G. (2024). Cancer Care Team Functioning during COVID-19: A Narrative Literature Review and Synthesis. Curr. Oncol..

